# Herniation Pits in Human Mummies: A CT Investigation in the Capuchin Catacombs of Palermo, Sicily

**DOI:** 10.1371/journal.pone.0036537

**Published:** 2012-05-02

**Authors:** Stephanie Panzer, Dario Piombino-Mascali, Albert R. Zink

**Affiliations:** 1 Department of Radiology, Trauma Center Murnau, Murnau, Germany; 2 EURAC - Institute for Mummies and the Iceman, Bolzano, Italy; Mayo Clinic College of Medicine, United States of America

## Abstract

Herniation pits (HPs) of the femoral neck were first described in a radiological publication in 1982 as round to oval radiolucencies in the proximal superior quadrant of the femoral neck on anteroposterior radiographs of adults. In following early clinical publications, HPs were generally recognized as an incidental finding. In contrast, in current clinical literature they are mentioned in the context of femoroacetabular impingement (FAI) of the hip joint, which is known to cause osteoarthritis (OA). The significance of HPs in chronic skeletal disorders such as OA is still unclear, but they are discussed as a possible radiological indicator for FAI in a large part of clinical studies.

In this paleoradiological study we examined a sample of mummies from the Capuchin Catacombs of Palermo, Sicily, by a mobile computed tomography (CT) scanner. Evaluation of the CT examinations revealed HPs in six out of 16 (37.5%) adult male mummies.

The first aim of this study was to compare the characteristics of HPs shown in our mummy collection to the findings described in clinical literature. Thereby CT evaluation revealed that their osseous imaging characteristics are in accordance, consisting of round to oval subcortical lesions at the anterior femoral neck, clearly demarcated by a sclerotic margin.

The second aim was to introduce HPs to the paleoradiological and paleopathological methodology as an entity that underwent a renaissance from an incidental finding to a possible radiological indicator of FAI in the clinical situation. As FAI plays an important role in the development of OA of the hip, which is a very common finding in human skeletal remains, HPs should always be considered in paleoradiological evaluation of hip joint diseases.

## Introduction

Paleopathology is the study of diseases and related conditions in skeletal and mummified remains that include the application of different methods and techniques and the use of various sources [1]–[3]. Since the first X-ray study of human and animal mummies, performed by König as early as 1896 [4], paleopathology has benefitted from the use of radiological methods and technical innovations in radiology, such as computed tomography (CT) and magnetic resonance imaging (MRI) by enhancing the possibility in diagnosing pathological conditions in ancient skeletons and mummies. Collaboration between anthropologists and musculoskeletal radiologists may facilitate solving diagnostic problems arising during analysis of ancient human remains, and radiological textbooks have become an important source in paleopathological studies [5]. Thereby, the contribution of musculoskeletal radiologists is not only to assist with diagnosis, but also to provide information on the clinical setting and relevance of diseases [6].

One of the most common described pathologies in ancient human remains is osteoarthritis (OA), or degenerative joint disease [7]. In studies of skeletal remains, OA of the hip is usually diagnosed by the presence of at least one of the following criteria: periarticular bone formation, subchondral bone resorption, and eburnation [2], [8]. While eburnation is a clear indicator of advanced OA, in both clinical and paleopathological studies other surface modifications, such as pitting or porosity, have been proposed to be directly related to OA [9], [10].

In paleoepidemiological studies, OA scores are widely used in order to reconstruct lifestyle, mobility and activity patterns in ancient populations with special emphasis on the biocultural context [11]–[15]. These studies are based on the assumption that activity, such as repetitive mechanical loading on muscles and joints, is the primary contributing factor [16]. This approach allows scholars to use osteoarthritic prevalence and severity in order to evaluate physical stress and specific activities in historic populations [12]. Nevertheless, most authors are aware of the complex etiopathogenesis of OA. Several other factors, such as sex, weight, nutrition, endocrine status and genetic influences may in fact contribute to the development of OA. Moreover, it has also been noted that anatomical variation can affect joints differently and influence the onset of OA. As an example, Reijmann and colleagues [17] showed that patients with acetabular dysplasia have a higher risk of hip OA.

Another condition associated with hip osteoarthritis is femoroacetabular impingement (FAI). In FAI, morphologic abnormalities of the proximal femur, acetabulum or both cause abnormal contact between the femur and acetabulum during motion, especially during flexion and internal rotation. This contact causes abnormal stress on the acetabular labrum and articular cartilage. The resulting degeneration, tearing of the labrum and damage to the adjacent acetabular cartilage can lead to OA of the hip joint [18], [19]. In the current clinical literature, minor osseous changes located in the femoral neck, or the so-called herniation pits (HPs), were mentioned in the context of FAI.

Herniation pits were first described by Pitt and colleagues in 1982 [20]. On anteroposterior (AP) radiographs of adult femoral necks, the authors identified well-delineated, round to oval radiolucencies in the proximal superior quadrant of the femoral neck, projected 5–15 mm below the superior neck margin, directly beneath the anterior cortex, bordered by a thin, organized sclerotic margin. Pitt and co-workers [20] generally recognized HPs as benign and incidental and believed that HPs, although sharing a common teleologic mechanism of soft-tissue herniation characteristic of some ganglia, are a different, unique entity.

Since this first definition of HPs, a number of case reports and reviews in clinical literature were published, describing especially the radiological findings in CT, MRI, micro-CT and bone scanning, the macro-pathology and histology, as well as the location and the prevalence of HPs [21]–[31]. In contrast to the early publications, in which HPs were generally described as an incidental finding [20], [22], more recent clinical studies regard them as a possible radiographic indicator for FAI [e.g.], [ 32]–[38].

In this study, we focus on the paleoradiological investigation of HPs in a sample of human mummies from the Capuchin Catacombs in Palermo, Sicily. Until now, HPs and FAI have not been widely taken into consideration in paleopathological or paleoradiological studies. Chhem and Brothwell [6] describe HPs, but classify them as normal variants, while Villotte and Knüsel [39] discuss the role of FAI with regard to osseous non-metric traits of the femoral neck. Therefore, the goal of our study was to introduce HPs to the paleoradiological and paleopathological methodology as a possible indicator of FAI.

As FAI plays an important role in the development of hip OA, which is a very common finding in human skeletal remains, we believe that HPs should be taken into account in future paleopathological studies.

## Materials and Methods

**Figure 1:**
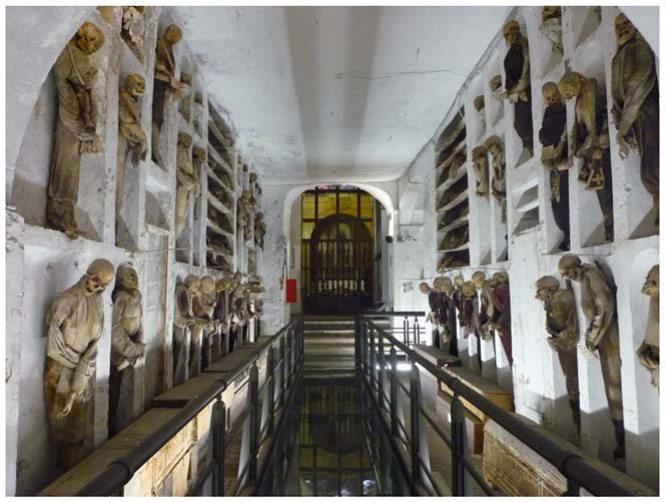
Image of one of the corridors of the Catacombs. Over eighteen hundred bodies, many of which still retain soft tissue, are displayed along the sides of the different corridors. Many of them are stored in wall niches or upon shelves while others lie in wooden coffins. Over time, bodies were grouped according to sex, age and occupation. Almost all mummies are clothed and wear hats, shoes or even gloves.

In December of 2010 we had the opportunity to examine a sample group of 26 mummies, including 16 adult males and 10 immature individuals preserved in the Capuchin Catacombs of Palermo. At the end of the 16^th^ century, the Catacombs were constructed as a burial site for deceased friars. Over the course of time, extensive subterranean corridors were carved out of massive deposit of tuff that underlies the Capuchin Church and Convent. The first mummified bodies were placed there in 1599, and the last ones in the early 20^th^ century [40], [41]. Today the Catacombs form an impressive site where over twelve hundred bodies, many of which still retain soft tissue, are displayed along the sides of the corridors (Figure 1). The Catacomb mummies of Sicily are mainly the result of a spontaneous-enhanced preservation mechanism. Shortly after death, bodies were taken to special preparation rooms, and laid on terracotta racks designed to allow draining of the body fluids and promote spontaneous desiccation of the cadavers. The rooms were then sealed for approximately one year, after which time the corpses were exposed to the air, washed with vinegar and dressed. Some bodies were also preserved with anthropogenic methods, such as briefly immersing the bodies in lime or by means of arterially injecting specific chemicals [40]–[43].

For the present study, only the adult sample group, consisting of 16 males dating from the late 18^th^ to the late 19^th^ centuries AD, was considered. The sample selection was mainly based on the accessibility and preservation state of the bodies. Furthermore, the short time availability of the mobile CT scanner only allowed a small number of mummies to be studied. Any available information on these subjects, such as age, identity, occupation and time of death was either determined through observation of the artifacts associated with the bodies and the coffins, the embalming technique employed, or gleaned from the death records present in the Archives of the Capuchin Convent and in the Municipal Archives of Palermo. At least six bodies from the sample group underwent a process of anthropogenic mummification.

The mummies were examined by a mobile 4-section CT scanner (Alliance Medical, Warwick, UK; LightSpeed Plus, GE Healthcare, Milwaukee, Wisconsin, USA) which was positioned in front of the Capuchin Church next to the entrance of the Catacombs. All examinations were obtained as whole body CT in helical technique with slice thickness of 1.25 mm, interval of 1.25 mm and pitch of 0.75 with 120 kV in a standard algorithm. Paleoradiological evaluation as well as performance of multi-planar reconstructions (MPR) was carried out at the Picture Archiving and Communicating System (ImpaxEE, Agfa HealthCare, Bonn, Germany) in the Department of Radiology by the first author who is also experienced in paleoradiology and musculoskeletal radiology.

HPs at the femoral neck were characterized by size, shape, margin, cortical breaks and Hounsfield Unit (HU) representing the density values in CT examinations. In case of mummies with HPs, MPRs of the respective hip joint were generated. The location of each HP was classified in the parasagittal plane in which the anterior, almost semicircular half of the femoral neck was divided into a superior and inferior portion and the transition zone between the two [37].

**Figure 2:**
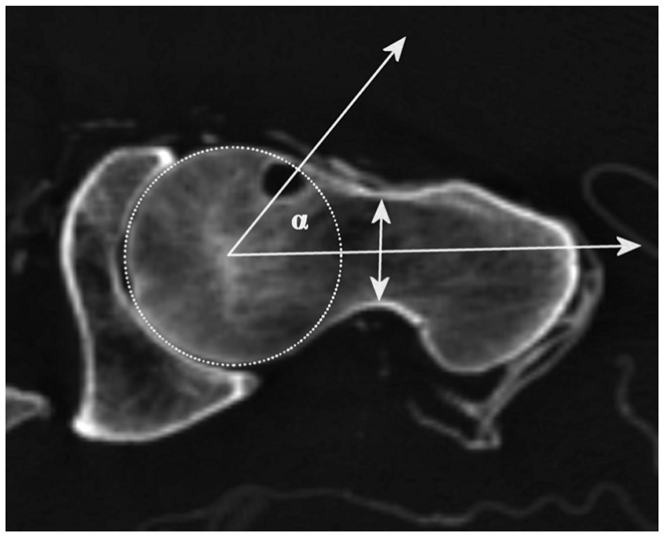
Measurement of the angle α. Paraaxial (parallel to the axis of the femoral neck) reformation CT image in case 3. The angle α was defined by a line between the centre of the femoral head and the anterior point, where the distance of the centre of the femoral head exceeds the radius of the surface of the femoral head and a second line between the centre of the femoral head and the centre of the femoral neck at its narrowest point.

To determine the femoral head-neck junction, the angle α introduced by Nötzli et al. [44] was measured on a plane parallel to the axis of the femoral neck passing through the centre of the femoral head. The angle α was defined by two lines: the first line between the centre of the femoral head and the anterior point where the distance of the centre of the femoral head exceeds the radius of the surface of the femoral head and the second line between the centre of the femoral head and the centre of the femoral neck at its narrowest point (Figure 2).

Degenerative disease of the hip joints containing HPs was evaluated by the assessment of osteophytes, subchondral cysts and eburnation. The criterion of joint space narrowing, which is used in the clinical situation, is not applicable to paleoradiology as soft tissues and joint alignment in mummies are known to change as a result of dehydration [6].

## Results

**Figure 3:**
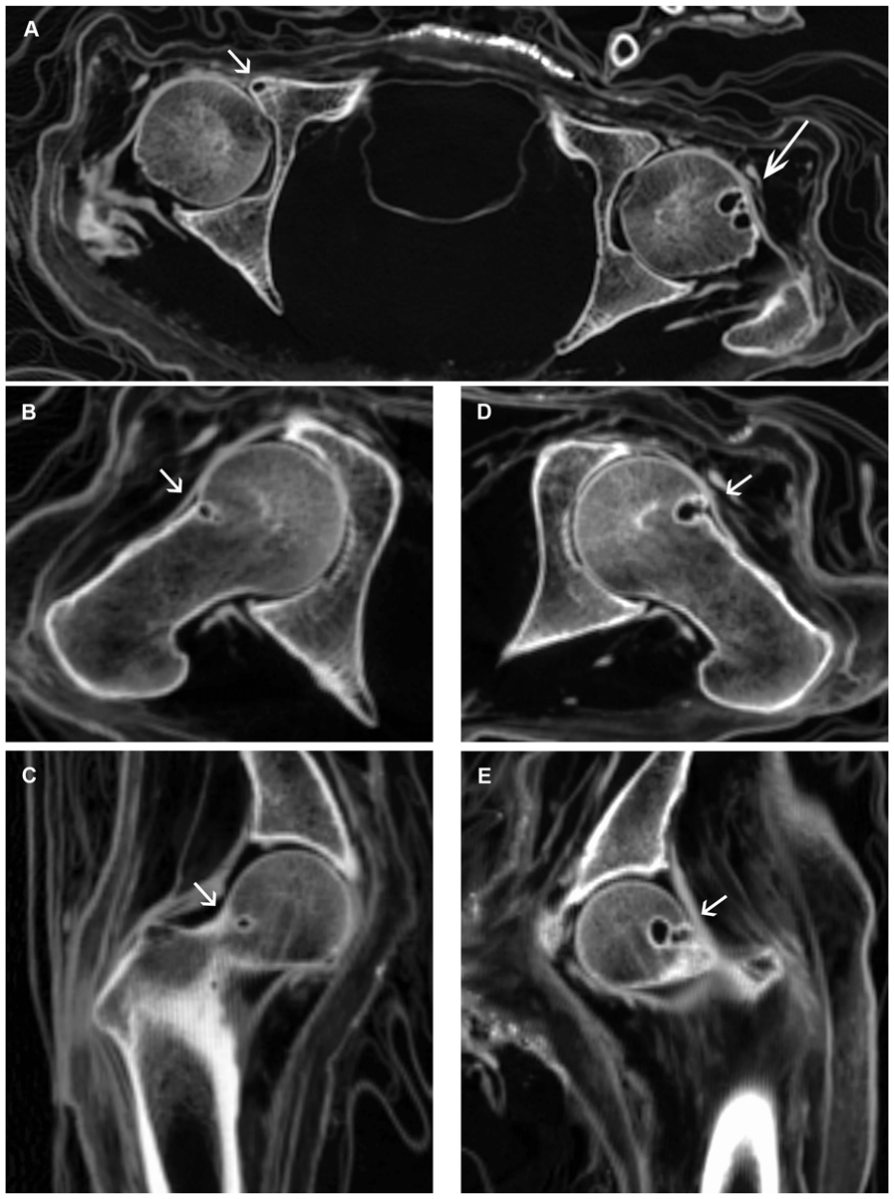
CT examination of case 2 with bilateral and multifocal HPs. (**A**) Axial CT slice illustrating a small subcortical/subchondral cyst inside the anterior border of the right acetabulum (short arrow) and multifocal HPs in the left anterior femur (long arrow). (**B**) Paraxial and (**C**) paracoronar CT reformation images of the right femur showing a small round HP with sclerotic margin (arrow). (**D**) Paraxial CT reformation image of the left femur demonstrating a HP with clearly visible cortical break (arrow). Note partial preservation of the joint capsule including labral structures. (**E**) Paracoronar CT reformation image of the left femur showing one round to oval HP medially and one round to oval lobulated HP laterally (arrow).

All investigated mummies showed very good preservation status of the skeletal structures, allowing precise assessment of morphological changes. In some mummies, preserved parts of the joint capsule and labrum were visible (Figure 3).

**Figure 4:**
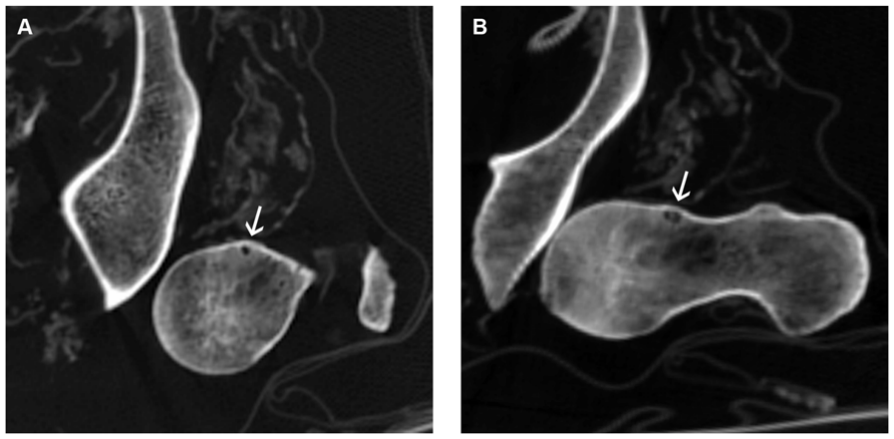
CT examination of case 6 with HP and a prominent anterior femoral head neck junction. Disarticulated left femur. (**A**) Axial CT slice illustrating a small round to oval HP (arrow). (**B**) Paraxial CT reformation image demonstrating the prominent anterior femoral head neck junction and the HP at the femoral neck (arrow).

**Figure 5:**
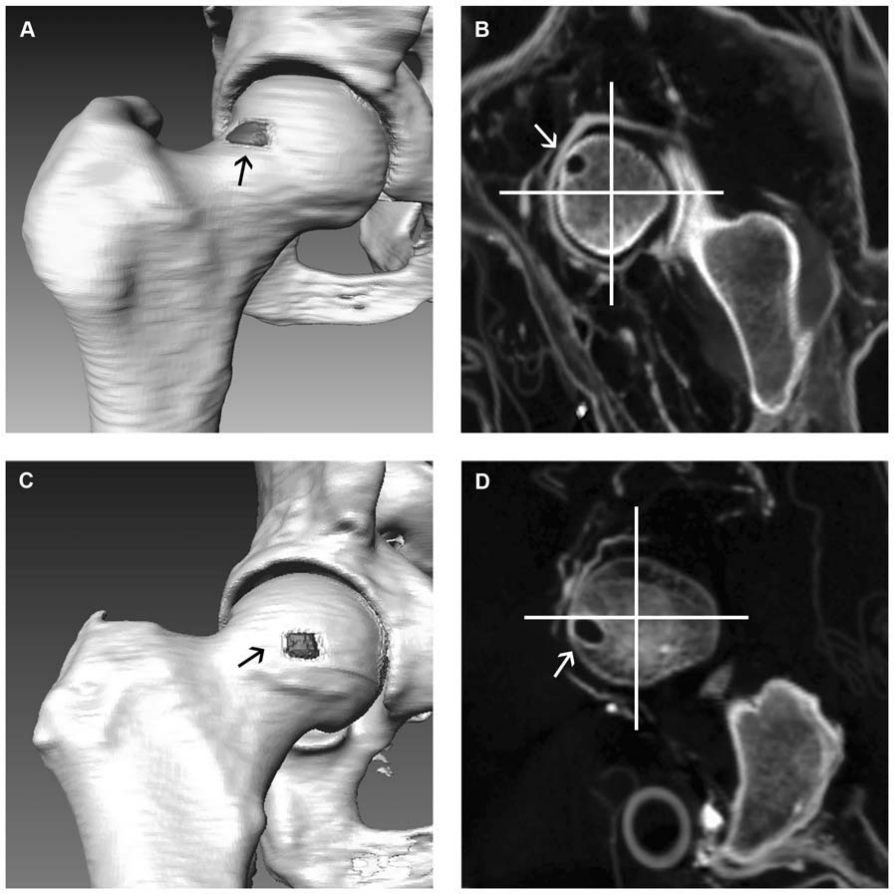
Location of HPs. Typical location of HPs in the superior portion of the anterior femoral neck (**A**, **B**) and less common location in the transition zone between the superior and inferior portion of the anterior femoral neck (**C**, **D**). (**A**, **C**) Modified three-dimensional CT reconstructions of living patients with HPs. (**B**, **D**) Parasagittal (perpendicular to the femoral neck axis) CT reformation images of case 2 and 3 with division into a superior (between 9 and 12 o'clock) and inferior (between 6 and 9 o'clock) portion.

CT examination revealed a total of 11 HPs in six out of the 16 investigated adult male mummies (Table 1), while no HPs were detected in the child mummies. According to the death records, one person died at the age of 56 years and another one at the age of 73 years. The remaining four cases were estimated to an age range of about 40 to 70 years based on the radiological appearance. Two mummies showed bilateral HPs as well as multiplicity in one femur of each case (Figure 3). Two mummies had a single HP in the left femur, and two had a single HP in the right femur. The femur of case 6 was disarticulated postmortem (Figure 4). The maximum diameter of the 11 HPs ranged from 3 to 13 mm with a mean of 7.4 mm. They were round or round to oval in shape and in three cases they were slightly lobulated. All HPs were demarcated by a complete sclerotic margin (Figure 3, Figure 4, Figure 5B, D). Cortical breaks were discernible in six HPs (Figure 3D). HU values measured within the HPs ranged from −763 to −980 with a mean of −908.8. All HPs were located below the growth plate of the femoral head, five in the superior portion of the femoral neck (Figure 5B) and six in the transition zone between the superior and inferior portion (Figure 5D). The angle α ranged from 33° to 68° with a mean of 42.4° (Table 2).

**Table 1:** CT-characteristics and location of HPs.

Case	1	2			3				4	5	6
Femur	right	right	left	left	right	right	right	left	left	right	left
**Size (mm)**	12	5	9	8	7	4	3	13	3	10	7
**Shape**	round-oval, lobul.	round	round-oval	round-oval, lobul.	round-oval	round	oval	round-oval	round-oval	round-oval	round-oval, lobul.
**Margin**	sclerotic	sclerotic	sclerotic	sclerotic	sclerotic	sclerotic	sclerotic	sclerotic	sclerotic	sclerotic	sclerotic
**Cortical Breaks**	-	-	1	1	-	1	-	1	1	1	-
**HU**	−873	−888	−958	−960	−948	−949	−893	−980	−763	−935	−850
**Location**	trans.	superior	superior medial	superior lateral	trans. medial	trans. middle	trans. lateral	trans.	superior	superior	trans.

HU Hounsfield Units.

lobul. lobulated.

trans. transition zone.

**Table 2:** Angle α of the femurs with HPs and signs of OA of the respective hip joint.

Case	1	2		3		4	5	6
Femur	right	right	left	right	left	left	right	left
**Angle α**	45°	33°	38°	38°	50°	38°	42°	68°
**Osteo-phytes**	slight acetabular	-	-	slight acetabular	sligth acetabular	moderate acetabular, slight femoral	slight acetabular	slight acetabular
**Cysts**	-	anterior acetabular border	-	-	-	acetabular roof	anterior acetabular border	-

Evaluation of OA of the hip joints with HPs (Table 2) revealed slight osteophytes of the lateral acetabular roof in cases 1, 3, 5 and 6 with additional slight osteophytes of the femoral head in case 6. Case 4 showed moderate osteophytes and small subchondral cysts of the acetabular roof as well as slight osteophytes of the femoral head. At the anterior border of the right acetabulum of case 2 and of case 5 respectively, one round to oval lesion with a well-defined sclerotic margin and a diameter of 4 to 5 mm was found subcortically/subchondrally, both representing small cysts (Figure 3A). The lesion in case 2 showed a cortical break. Eburnation was not found in any case.

As additional findings which did not affect the hip joint directly, case 1 showed a subcortical lobulated lesion with well-defined sclerotic margins and osseous septa in the ventrolateral aspect of the right acetabulum. The maximum diameter was 2.7 cm. The lesion revealed a cortical break. In case 3, a lobulated lesion with well-defined sclerotic margins and a thin peripheral osseous septum was detectable subcortically/subchondrally in the ventrolateral part of the acetabular roof. The maximum diameter was 2 cm and a cortical break was visible. In both mummies the described lesions were monostotic. Both lesions represent benign tumors or tumor-like lesions, e.g., simple bone cysts or fibrous dysplasia.

## Discussion

We found HPs in six out of 16 (37.5%) adult male mummies respectively in eight out of 32 (25%) femora. Prevalence of HPs in clinical studies ranged from 2.6% (four out of 152 patients) [26] to 42.5% (85 out of 200 patients) [37], respectively 4.6% (one out of 22 femora) [45] to 35.4% [35] if referring to the single femora. Thereby the investigated collectives differed mainly between normal adults [20], [22], incidental collectives [24], [37] and clinically symptomatic patients with FAI [35], [45]–[48]. Collectives with normal adults and incidental collectives ranged from 4.2% [22] to 42.5% [37] referring to patients. In clinical literature, the age of patients ranged from 22 to 62 years in case reports on HPs [21], [23], [25]–[30], and from 14 to 96 years in studies on FAI mentioning HPs (37,45).

In this study, we were only able to investigate a small collection of exclusively male adult mummies, making a comparison with clinical studies difficult. Nevertheless, the prevalence and age we found in the mummies of the Capuchin Catacombs lies inside the range of published data from the clinical literature. Bilateral HPs and multiplicity of HPs in a femur as shown in two of our investigated mummies are also known findings [37].

The size of HPs in our collection, with a mean diameter of 7.4 mm, was comparable to the average size given in clinical publications. According to Pitt et al. [20] the maximum diameter of HPs is usually less than 10 mm. Studies based on high case numbers report an average diameter of 5 mm (range 3–15 mm) on x-rays [47] and 5.3 mm (range 2–11 mm) in CT examinations [37].

The usual appearance of HPs in clinical CT examinations is a round to oval, sometimes lobulated, subcortical lesion containing connective tissue and fat, surrounded by a sclerotic margin. Cortical perforations or breaks are frequently discernible [20], [23], [28], [37]. The CT appearance of the HPs detected in our mummies was in full accordance with the described osseous characteristics. The density measured inside the HPs (HU values ranging from −763 to −980) was significantly lower than that generally measured in studies on living patients, in which density (HU) values were ranging from −79 to 380, as described by Panzer et al. [37] in a large-scale study. This can be explained by the fact that the HPs in the studied mummies are mainly filled with air as at least most of the fat and connective tissue inside the HPs has degraded over time.

The typical location of HPs is the superior portion of the anterior femoral neck [20], while in some cases HPs are located in the inferior anterior femoral neck [28], [37] or at the transition zone between the superior to inferior anterior portion [37]. In the mummy sample group HPs were found in the superior portion of the femoral neck as well as in the transition zone.

Pitt et al. [20] showed that HPs are formed by herniation of soft tissues through erosions or perforations, which result from abrasive action of the overlying hip capsule. They believed that the HP is a unique entity. In recent publications, it is postulated that HPs are intraosseous ganglia [31], [47]. Furthermore, the results of the study of Leunig et al. [47] indicated that there is a close spatial relationship between HPs and the site of FAI, favoring the theory that flexion-induced pressure causes the production of HPs.

In differential diagnosis, HPs must be differentiated from subchondral cysts that occur in OA. The latter are usually located in the central area of the joint surface where the stress of weight bearing has the highest impact [49]. In contrast to subchondral cysts, HPs are predominantly localized at the peripheral side of the anterior femoral neck. Further differential diagnoses that should be taken into account are osteoid osteomas [20], [22], [25], [28], [30], [47], malignant diseases such as atypical osseous metastasis [22], [29], [30], [47] and lymphoma [47], inflammatory disease such as Brodie's abscess [20], [22], [25], [29], [30] or iliopsoas tendinitis, -bursitis [30] as well as focal avascular necrosis [22], [30], focal osteoporosis, unspecific trabecular restructuring and degenerative changes [31]. Nevertheless, HPs should be clearly differentiable from these by means of their typical CT appearance as round to oval subcortical/subchondral lesions with encircling sclerotic margin and their typical location at the anterior femoral neck.

In early clinical publications, HPs were generally reported as an incidental finding [20], [22], whereas in current radiological and orthopedic literature they are mentioned within the context of FAI and OA of the hip [38]. The etiology of OA of the hip has long been considered primary (presuming some underlying abnormality of the articular cartilage) or secondary (e.g. due to congenital or developmental deformities). Recent findings support a hypothesis that the so-called primary OA has also to be regarded as secondary OA due to tenuous developmental abnormalities in which the underlying mechanism is FAI [19]. Four decades ago, Murray [50] proposed the term “tilt deformity of the femoral head” in order to describe an abnormal relationship of the femoral head to the femoral neck as a slight anatomical variation. Anteroposterior radiographs suggested a mild degree of the deformity commonly occurring after a minimal slipped capital femoral epiphysis [19]. Stulberg et al. [51] introduced the term “pistol-grip deformity” for a femoral head and neck configuration, consisting of a flattening of the usually concave surface of the lateral femoral neck and a bump on the anterolateral surface of the femoral neck visible on AP radiographs. Two different types of FAI have been described. Cam-type FAI, which is more prevalent in young male patients, is caused by an offset pathomorphology between the head and neck of the femur that leads to an outside-in delamination of the acetabulum. Pincer-type FAI, which is more prevalent in middle-aged women, is caused by a more linear impact between a local (retroversion of the acetabulum) or general overcoverage (coxa profunda/protrusion) of the acetabulum. Most hips however show a mixed FAI pattern with cam predominance [19], [52].

In the numerous ongoing studies on FAI, a tendency towards the association of HPs to cam-type became apparent [19], [53]–[55]. The use of CT and MRI allowed the introduction of new measurements for FAI in any desired plane and even for three-dimensional analysis [55]. A relatively simple measurement for the assessment of pathomorphological changes in the femoral head and neck in CT and MRI examinations is the determination of the angle α [44]. In this study, we have chosen this measurement of the anterior position of the femoral head-neck junction as it represents, together with the anterosuperior position, the most frequent location for FAI [19], [56].

In the mummy samples, one out of a total of 8 femora with the presence of HPs showed an angle α larger than 50° (case 6, angle α 68°, see Table 2), which is the cut-off angle in the description of [44]. In another femur (case 3) an angle α of exactly 50° was measured. We can thus provide evidence for one femur showing the typical pathomorphology of the head neck junction indicating cam-type FAI and another one that has to be regarded as a borderline case. The remaining 6 femora did not show any abnormal anatomy of the head neck junction based on the measurement of the angle α.

Slight degenerative changes of the hip joint were found in four cases, including the two femora with an angle α of 50° respectively 68°. In these two cases, the prominent head neck junction could have led to FAI resulting in mild degenerative disease. One case with moderate degenerative changes had a normal angle α of 38°. In this case the development of OA cannot be directly linked to a pathological anatomy of the proximal femur, despite the presence of a HP.

In paleopathology, OA or degenerative joint disease is a very common and well-described disease [3], [49], [57]. It is widely used in paleoepidemiological studies as a main indicator for mechanical stress and activity in ancient populations [12]–[14]. Although the complexity of the pathogenesis of OA has been noted by some authors [58]–[60], little attention has drawn to the role of morphological alterations in the etiology of OA of the hip and OA in general [7]. It is important to note, that in the clinical literature, OA of the hip is mainly linked to morphological deformities, such as developmental dysplasia of the hip [61]. In more recent work, FAI was brought out as an important mechanism for the development of hip OA [19], [35]. Therefore, more focus should be placed on abnormal hip morphology as a cause for OA in paleopathological studies. In radiological but also morphological examinations of human remains, the above described angle α could be easily determined as an indicator for FAI. As HPs can apparently be linked to FAI and OA of the hip, they should be recognized and documented as a finding in the paleoradiological evaluation of hip joint diseases, in addition to the well-established criteria of degenerative disease like eburnation, osteophytes and subchondral cysts. In skeletons or mummies, it is rarely possible to assess labral or chondral structures, but as shown in case 2 and 5 of our study, the small subcortical/subchondral cysts in the peripheral part of the anterior acetabulum can serve as indicators for chondral and labral tears. In particular, chondral abnormalities situated peripherally at the labral-chondral transition zone lead to changes in the adjacent subchondral bone including the development of subchondral cysts [18], [48], [62]. Labral tears predispose toward extraosseous ganglia formation with splitting of the labrum and acetabular cartilage. This allows synovial fluid to penetrate into the subchondral bone, leading to a subchondral cyst formation [33]. The acetabular findings in case 1 and 3, representing benign tumors or tumor-like lesions, are most probably not associated with pathologies of the hip joint.

As far as possible, CT should be favored over conventional X-ray for the investigation of HPs in paleoradiological studies. CT scans allow a much better imaging of skeletal structures without any superimposition and are independent of the positioning during the examination. As an example, in our own previous study one of the mummies (case 1) was examined by means of digital X-ray [42]. The HP inside the right femur of this case was not detectable, despite a recent re-evaluation of the X-ray images.

The CT investigation of the mummy collection in this study showed that HPs could clearly be diagnosed in mummies and that the osseous imaging characteristics were comparable to those described in clinical literature. HPs can be identified in CT scans as round to oval subcortical/subchondral lesions at the anterior femoral neck, which is clearly demarcated by an encircling sclerotic margin. In the course of the last 30 years, the perception of HPs underwent a renaissance in clinical, radiological and orthopedic literature from an incidental finding to a possible radiological indicator for FAI, which is known to cause OA of the hip joint. OA of the hip joint is a very common observation in paleopathology and the discussed clinical relevance of HPs should also be addressed in paleoradiology. We believe that HPs should be detected and documented in paleoradiological diagnosis of hip joint disease and even subtle morphological changes of the hip joint, indicating FAI, should be taken into consideration as a cause for this condition.
